# Psychological interventions for managing postpartum psychosis: a qualitative analysis of women’s and family members’ experiences and preferences

**DOI:** 10.1186/s12888-019-2378-y

**Published:** 2019-12-19

**Authors:** R. Forde, S. Peters, A. Wittkowski

**Affiliations:** 1 Division of Psychology and Mental Health, School of Health Sciences, Faculty of Biology, Medicine and Health, The University of Manchester, Manchester Academic Health Science Centre, Zochonis Building, Brunswick Street, Manchester, M13 9PL UK; 2 Greater Manchester Mental Health NHS Foundation Trust, Manchester, UK

**Keywords:** Perinatal mental health, Psychotic disorders, Childbirth, Psychology, Psychosocial, Intervention

## Abstract

**Background:**

Postpartum psychosis is a rare, yet severe disorder, in which early identification and immediate intervention are crucial. Despite recommendations for psychological input, little is known about the types of psychological intervention reported to be helpful. The aim of this study was to explore the experiences, needs and preferences for psychological intervention from the perspective of women with postpartum psychosis and from the perspective of family members.

**Methods:**

Thirteen women and eight family members, including partners were interviewed. The data from these semi-structured interviews were audio-recorded, transcribed and inductively analysed using thematic analysis.

**Results:**

Twelve subthemes were identified and then organised around three main themes: 1) *Seeking safety and containment,* 2) *Recognising and responding to the psychological impact* and 3) *Planning for the future*. These themes highlight the temporal element of recovery from postpartum psychosis, because women’s psychological needs and preferences changed over time. Emphasis was initially placed on ensuring safety, followed by a need to connect, process and adjust to their experiences. Additional needs were reported by women and family when planning for the future, including managing the fear of relapse and help to reach a decision about future pregnancies.

**Conclusion:**

The results illustrate a range of areas in which psychological intervention could be delivered to facilitate and enhance recovery. Further research is needed to develop meaningful and effective psychological interventions and to investigate the most appropriate timing for this to be offered.

## Background

Postpartum psychosis is a relatively rare, yet severe mental health disorder affecting between 0.89 and 2.6 in 1000 women following childbirth [1]. Symptoms including hallucinations, delusions, mania and depression typically present with a sudden onset during the first postpartum week, but an increased risk remains during the first 90 days [2, 3]. Women with a history of bipolar disorder, previous postpartum disorders or increased maternal age are at a higher risk of developing postpartum psychosis; however, for almost 50% of women no risk factors have been identified [4].

As poorly managed episodes of postpartum psychosis can increase the risk of maternal and infant accidents and maternal and first-degree relative suicide [5], early identification and access to acute intervention is crucial [6]. Postpartum psychosis is a ‘psychiatric emergency’ (p. 411 [7];) requiring immediate assessment and usually a hospital admission to manage the acute symptoms [8]. Women should be admitted to a Mother and Baby Unit (MBU) for specialist treatment and to support the development of the mother-infant relationship [8–10]. However ‘patchy’ service provision means this is not always possible (p. 148 [11];). In the United Kingdom, the need for appropriate and timely intervention has been recognised and relevant mental health pathways have been developed including improved access to community perinatal mental health teams and additional MBU provision [12].

The short-term clinical outcomes are promising and symptoms of postpartum psychosis are typically limited in time [13]. However, the experience of postpartum psychosis can be very stressful for the woman and her family [14] and women are at increased risk of subsequent postpartum and non-postpartum episodes resulting in hospital readmission [15]. A systematic review of 15 qualitative studies, capturing the views of women and family members, concluded that long-term recovery from postpartum psychosis is a complex process in which women need to process and integrate their experiences, whilst transitioning to their new role and navigating a profound sense of loss and feelings of guilt [16]. Moreover, psychological problems can continue long after the acute symptoms of postpartum psychosis resolve. At nine months postpartum, it was found that women reported significantly more symptoms of depression and generalised anxiety compared to a matched reference group and 25% of women reported impairment in their psychosocial functioning [14].

It is therefore important that women continue to receive support and appropriate intervention after the acute symptoms of postpartum psychosis resolve. Practical and social interventions reported to be helpful include support to enhance the mother-baby-interaction and access to information and support groups to help normalise their experiences and reduce feelings of isolation [17–19]. Psychological intervention is also recommended [20]. However, to date no study has specifically investigated the range and type of psychological support that has been or could be beneficial for women experiencing postpartum psychosis. This contrasts with the significant evidence base for other perinatal mental health disorders (e.g., postnatal depression and anxiety [9, 21, 22]) and first episode psychosis in which Cognitive Behavioural Therapy (CBT) and family interventions are recommended to improve coping skills and reduce relapse rates [23–25].

Furthermore, families and partners have been found to play a pivotal role and their support is interwoven through each stage of the woman’s recovery, including the recognition of symptoms and provision of help to access services and maintain recovery [26–28]. Family members are considered a central resource during care and treatment planning [27, 28], however differences have been found in family members ability to cope and provide the level of support needed to manage at home [18]. There has been limited empirical investigation into their experiences in supporting women and their views of psychological treatment needs for the women they are supporting. Their views are pertinent, especially in relation to the acute phase; a time in which women often report gaps in their memory and are more dependent on others [19].

It is therefore important that research is conducted to identify and understand women’s psychological needs and to inform the future development of complex interventions [29, 30]. Thus, the aim of this study was to explore and understand the psychological needs of women with postpartum psychosis, from the perspective of women and family members and to investigate their experiences and preferences for psychological intervention.

## Method

### Design

This study used a qualitative design to investigate the views and experiences of women with direct experience of postpartum psychosis and of family members, including partners. Participant involvement was sought on an individual basis and interviews conducted separately. Participants were recruited through convenience sampling, guided by the principles of purposive sampling with the aim to capture a more diverse range of experiences and achieve greater variation within the sample [31, 32]. Ethical approval for the study was granted by the National Health Service (NHS) Research Ethics Committee (Ref: 18/NW/0404), Health Research Authority and the Greater Manchester Mental Health Trust Research and Innovation department.

### Inclusion and exclusion criteria

Participants were eligible for inclusion if they were aged 18 years or above, deemed to have capacity and they or their relative reported to have received a diagnosis of postpartum psychosis for experiences that occurred ‘up to 3 months after childbirth’ in accordance with The National Institute for Health and Care Excellence (NICE) guidelines (p. 28 [9];). In order to capture a diverse range of experiences and to gather data about how the individual’s beliefs and strategies had developed over time no limitations were applied with regards to ‘time since onset’. A broad definition of family was applied and participants were eligible to take part if they reported to have had previous, subsequent, or no other pregnancies or mental health difficulties.

Due to the reported nature of postpartum psychosis as being distinct to childbirth [33], participants were excluded if they had an existing diagnosis of schizophrenia. Participants were also unable to participate if they were currently experiencing acute psychotic symptoms, deemed unable to provide informed consent, or were not sufficiently proficient in English to complete an interview conducted in English.

### Data collection

Recruitment took place across England and Wales; through the charity Action on Postpartum Psychosis (APP), a Mother and Baby Unit (MBU) and a community perinatal mental health team between August and December 2018. Participants interested in taking part were encouraged to contact the lead researcher, who provided further information. Interviews were offered at the participants’ convenience and were conducted over the telephone or at a location of their choice, thereby providing access to a greater range of participants [34]. Informed written consent, demographic information and a brief questionnaire, pertaining to diagnosis and treatment was completed by all participants prior to the interview.

Participants were interviewed using a semi-structured interview topic guide that was developed by the researchers in line with the study aims and following a review of the relevant literature (e.g., [18, 35]). Questions focused on participants’ experiences of postpartum psychosis, including symptoms observed, interventions and support accessed, the role of the family and additional psychological needs. All interviews were audio-recorded using an encrypted device and the majority transcribed verbatim by the lead researcher (62%). All remaining interviews (38%) were transcribed by a paid member of staff at the University of Manchester, experienced in transcription. Accuracy and familiarisation with the data were enhanced by listening to the audio recordings again and editing the transcripts. All anonymised transcripts were entered electronically and managed using NVIVO 12 qualitative data management software [36].

### Data analysis

The data were analysed using thematic analysis [37]. Thematic analysis allows for the provision of a rich and detailed account that captures similarities and differences across multiple perspectives. It also provides a flexible method for the identification of themes and patterns across data that is ‘independent of theory’ (p. 5 [37];). This was deemed important because there is limited theory and pre-existing research on experiences and psychological intervention for postpartum psychosis. Hence the coding was exploratory, data driven and the analysis applied inductively [38, 39]. Analysis followed Braun and Clarke’s [37] six key stages (see Table 1) using an iterative process, in which the researchers frequently moved between stages. As the aim of the research was primarily focussed on women’s psychological needs, the women’s accounts were coded first, followed by those of family members and constant comparisons were made within and between these accounts to ensure similarities and differences were captured. The final analysis was completed at a latent level, going ‘beyond’ the semantic meaning, in order to identify more implicit meaning (p. 252 [40];). Finally a thematic structure was developed that captured all relevant meanings in relation to the research aims.

**Table 1:** Analytical stages of Thematic Analysis used

Stage of Thematic Analysis [37]	
1	Familiarisation and immersion in the data; including transcription and repeated reading of the data, looking for patterns and meaning.
2	Generating initial codes that appeared interesting and organising the data into meaningful groups at a semantic level. All data was coded at this stage.
3	Searching for themes and re-focusing the analysis at a broader level. Considering how codes may combine to form an overarching theme.
4	Reviewing and re(defining)the themes at a latent level, returning to the raw data, discussing with the research team and modifying/merging themes as necessary to develop a thematic map.
5	Defining and naming themes and creating a consistent and coherent ‘story’.
6	Reporting the outcomes and linking the themes to the research question.

### Position of the researchers

The lead researcher was a clinical psychologist in training and the study formed part of her doctoral programme. The project was informed by her experience of having previously worked clinically with adults experiencing psychosis. She was interested in the unique experience of psychosis following childbirth and in systemic approaches which influenced her decision to seek families’ perspectives alongside the women’s experiences. The other two researchers, both of them mothers, have expertise in health and clinical psychology and experience of conducting qualitative research on lived experiences of mental health disorders, including postpartum psychosis. One of them also has extensive clinical experience of postpartum psychosis through her work on an MBU. Whilst this familiarity is a strength of the research because it provided a deeper understanding on the topic, efforts were taken to understand prior assumptions and analytic preconceptions brought to the analyses by the team, including use of a reflective log and frequent discussion within the research team.

### Rigour

Several techniques were utilised in line with existing qualitative guidelines for rigour [41, 42]. This included: 1) the lead researcher maintained a reflective log throughout to capture initial thoughts and ideas and consider the impact of her own role, assumptions and personal values [43]; 2) rich detail regarding the participants were recorded to facilitate analysis and support the reader to draw their own judgements; 3) credibility checks were completed during data analysis and interpretation, using researcher triangulation and external peer feedback was sought during theme development [42, 44]; 4) a thematic structure was developed to capture the relationship between themes and constant comparisons were made across the sample and 5) an audit trail, supported by the use of NVIVO 12 software [36], was maintained to provide transparency and enable scrutiny around decision-making and theme development [42, 43].

## Results

### Sample

In total, twenty-one participants were recruited through APP (*n* = 20) and an MBU (*n* = 1). An additional 11 individuals expressed interest but did not participate because they had not received a diagnosis of postpartum psychosis (*n* = 4), did not respond to follow up contact from the lead researcher (*n* = 3) or recruitment had ceased (n = 4). The final sample comprised 13 women and eight family members. Five of the family members were related to women who had taken part in the research, meaning that 16 unique episodes were captured. To maintain confidentiality, no comparisons are drawn between these family relations in the analysis [44]. All family members described themselves as involved in the woman’s life during her postpartum psychosis. Interviews were conducted face-to-face (*n* = 11) or over the telephone (*n* = 10) and interview length ranged from 36 min to 109 min (Mean = 62 min).

### Sample characteristics

Full participant characteristics are provided in Table 2. One woman had a prior diagnosis of bipolar disorder and three women were subsequently diagnosed with bipolar disorder. Three participants reported two episodes of postpartum psychosis. In this instance, both episodes were explored during the interview. All women and family members described themselves as White British and 19 were married or in a relationship. Sixteen of the participants were in employment and 17 attended further education. Women ranged in age from 25 to 44 years and their most recent onset of postpartum psychosis occurred between three months and 23 years ago (Mean = six years). Family members comprised four parents, three partners and one sibling. Family members ranged in age from 35 to 65+ years and the most recent episode of postpartum psychosis of their family member occurred between one year and 12 years ago (Mean = five years).

**Table 2:** Overview of participant characteristics

**Participant characteristic: Women with direct experiences of postpartum psychosis**								
**ID**	**Age range**	**Highest level of education**	**Work status**	**Relationship status**	**Child-ren**	**Time since onset**	**Treatment provider**	**Mental health history**
**W1**	25–34	High school	Part-time	Relationship	1	1–3 years	MBU, general psychiatric, home treatment, perinatal team	Depression, anxiety before and after PP
**W2**	35–44	University	Part-time	Relationship	2	12 years +	MBU, general psychiatric, home treatment	Depression, anxiety after PP
**W3**	25–34	University	Self-employed	Relationship	2	0–6 months (Most recent)	First managed at home, second MBU, home treatment	Depression after PP
**W4**	35–44	University	Self-employed	Relationship	2	6–12 months	MBU (readmitted), perinatal team	Depression, anxiety before and after PP
**W5**	45–54	University	Self-employed	Relationship	2	12 years + (Most recent)	General psychiatric for both, MBU second	None (Bipolar diagnosis given but does not relate to this)
**W6**	35–44	Training	Part-time	Relationship	2	3–6 years	General psychiatric	None
**W7**	35–44	College	Un-employed	Relationship	2	1–3 years	MBU	Bipolar disorder
**W8**	35–44	University	Part-time	Relationship	1	1–3 years	MBU (readmitted), home treatment, perinatal team	Panic attacks before PP. Depression, anxiety after PP
**W9**	35–44	Post-graduate	Part-time	Relationship	2	3–6 years	A&E, no immediate follow up.	None
**W10**	45–54	Post-graduate	Full-time	Single	1	9–12 years	None	Depression, anxiety before PP. Bipolar after PP
**W11**	25–34	University	Full-time	Relationship	1	3–6 years	MBU, general psychiatric, home treatment.	None
**W12**	45–54	College	Part-time	Separated	1	12 years +	General psychiatric. Later admitted to MBU.	Bipolar after PP
**W13**	35–44	Post-graduate	Part-time	Relationship	1	6–9 years	MBU, general psychiatric, community mental health team, IAPT	Depression, anxiety after PP
**Participant characteristic: Family members of people with direct experiences of postpartum psychosis**								
	**Age**	**Education**	**Employment**	**Relationship status**	**Child-ren**	**Time since onset**	**Treatment**	**Relationship**
**FM14**	65+	University	Retired	Relationship	2	1–3 years	MBU (readmitted) home treatment, perinatal team	Parent
**FM15**	35–44	College	Full-time	Relationship	2	3–6 years	A&E, no immediate follow up	Sibling
**FM16**	65+	High school	Retired	Relationship	4	12 years +	MBU, general psychiatric, Intensive home treatment	Parent
**FM17**	35–44	College	Full-time	Relationship	4	1–3 years	MBU	Partner
**FM18**	65+	College	Retired	Relationship	Not stated	12 years + (Most recent)	General psychiatric for both, MBU second	Parent
**FM19**	65+	High school	Retired	Relationship	3	6–9 years	MBU, general psychiatric	Parent
**FM20**	35–44	College	Part-time	Relationship	1	1–3 years	MBU (readmitted) home treatment, perinatal team	Partner
**FM21**	35–44	College	Full-time	Relationship	1	1–3 years	MBU, general psychiatric	Partner

### Experience of mental health and psychological intervention

As shown in Table 3, Women and family members reported a 94% hospital admission rate. Most women were initially admitted to a general psychiatric unit (56%) and subsequently transferred to an MBU, totalling 81% MBU admission. Two women were readmitted to an MBU due to subsequent depression. During their experience of postpartum psychosis, almost half of the women were under the care of either a community perinatal mental health team (25%) or community mental health team (19%). Most (69%) had accessed some form of psychological input, including CBT (24%), counselling (19%), psychotherapy (12%) and Eye Movement Desensitisation and Reprocessing (EMDR; 6%). Four had sought this input privately, either self-funded or through their work. Participants varied as to how useful or comprehensive they had found these interventions.

**Table 3:** Types of intervention offered, across 16 unique episodes

Reported intervention		Total^a^
Immediate intervention	MBU	13 (81%)
	General Psychiatric Unit	9 (56%)
	Home Treatment team	5 (31%)
	General Hospital	1 (6%)
	ECT	2 (12%)
	Total hospital admission	15 (94%)
Mental health provision	Community perinatal mental health team	4 (25%)
	Community mental health team (CMHT)	3 (19%)
	Total under secondary care	7 (44%)
Psychological input	CBT – accessed CBT – referral, but not accessed due to reported delays	2 (12%) 2 (12%)
	CBT for anxiety group	2 (12%)
	Access to Psychologist within community perinatal team	4 (25%)
	EMDR	1 (6%)
	Psychotherapy (two accessed privately)	2 (12%)
	Counselling (two accessed privately)	3 (19%)
	Total offered/accessed formal psychological input	11 (69%)
Other input accessed	Alternative therapy (Acupuncture, hypnotherapy)	2 (12%)
	Art therapy	2 (12%)
	Online self-help for managing Bipolar disorder	1 (6%)

^a^Some participants accessed more than one intervention hence numbers do not add to 100%

### Findings from the thematic analysis

**Fig. 1:**
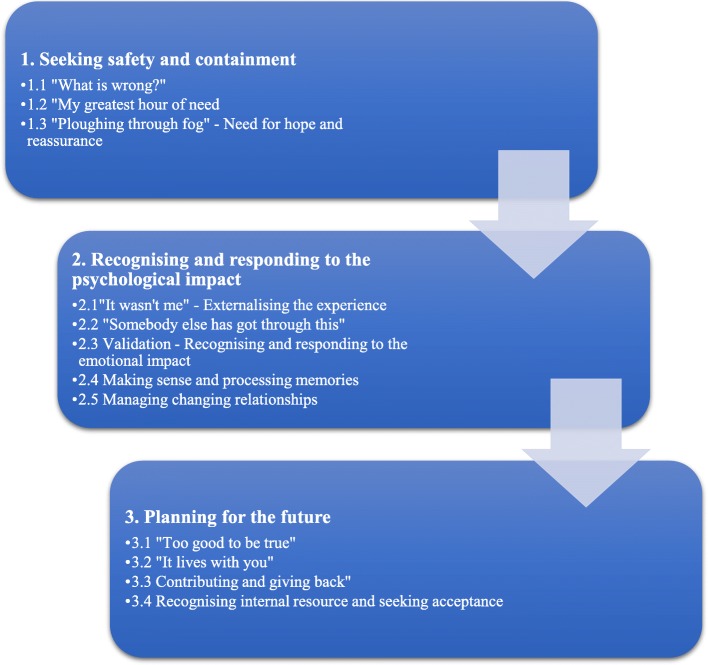
Thematic structure – main themes and subthemes as reported as by women and family members

As illustrated in Fig. 1, 12 subthemes were identified and then organised around three core themes: 1) *Seeking safety and containment*, 2) *Recognising and responding to the psychological impact* and 3) *Planning for the future*. The themes revealed recovery to be a complex process, with a unique temporal element, which commenced during the acute phase of postpartum psychosis but continued far beyond the initial episode. Transition towards recovery incorporated multiple psychological needs that had to be met to facilitate change and promote wellbeing, highlighting the importance of long-term recovery. Because of the overlap found across women’s and family members’ reported experiences, the thematic structure was developed from across the data corpus of women and family members. Each theme is described below and additional illustrative quotes can be found in Table 4.

**Table 4:** Additional illustrative quotes

**Theme 1: Seeking safety and containment**	
**1.1: “What is wrong?”**	
*“Ye it was a big, ye unexpected, no previous unwellness, no, no fore warning because it’s so sudden, and just, ye, it, umm the unravelling meant a complete loss of functioning really”* (Woman 9). *“I just sort of sat on the floor with her and tried to, tried to talk to her, as I would if she was the xx I love and know, erm, but it was very difficult, because, she was obviously very, very ill”* (Family member 15). *“These people are much cleverer and know much more about it than I do, so to hear it from somebody who knows what they’re talking about … somebody that you trust to give you the right answer, you know, says something, you think right, that’s what going to happen then”* (Family member 21).	
**1.2: “My greatest hour of need”**	
*“I remembered that I could trust her and I said ‘oh will you promise me something, that you’ll stay by my side and make sure they don’t kill me’. I did drift off to sleep at one point and I woke up and she was sat by my side and that meant so much having her there”* (Woman 12). *“I’d met up with the crisis team the next morning, but they said, no she’s fine.. and then my husband went in afterwards and he was, ‘actually she’s not fine, that’s not how she is’”* (Woman 13).	
**1.3 “Ploughing through fog” - Need for hope and reassurance**	
*“Umm, we did umm, mindfulness, so they would have a weekly session where we would do some mindfulness but I also had access to talk to a psychologist and just umm, building relationships with the staff who were there and just being able to share your thoughts and feelings with them, not in a, not in a labelled way, not in a CBT or, you know whatever, but just in a, in a supportive environment of being able, of them being there and being able to just be a friend and be umm, be a support as well”* (Woman 4). *“Erm, so we just really all sort of, muddled through, doing the best we could just trying to be as supportive as we could, and, talking amongst ourselves, helping each other. We did find, once we did get to the Mother and Baby unit that that was much better because, there was, you could always find somebody you could have a little chat to, and you could do it unobtrusively sort of thing”* (Family member 19).	
**Theme 2: Recognising and responding to the psychological impact**	
**2.1: “It wasn’t me” - Externalising the experience**	
*“Even over a long period of time that’s [memory] never come back but hmm obviously from my ex describing it and from the doctors as well, because you learn about these things through other people when you’re not quite with it yourself, but it was like I wasn’t even there. I was kind of, it wasn’t me at all. I could have killed everybody you know, in the whole town and I wouldn’t have been aware of it.. you know”* (Woman 5). *“I suppose once, I’d sort of looked into, into it [postpartum psychosis], that it, it explained what had been going on, and it certainly explained obviously the, the sort, you know seeing things, hearing things side of it, and, but yeah, it also explained sort of how normal behaviour had sort of turned into something that was disruptive”* (Family member 20).	
**2.2: “Somebody else has got through this”**	
*“Erm, but I remember, well, the one thing that sticks in my mind is, erm, I was, obviously, was looking at information about postpartum psychosis, ‘cos I found a charity, erm, and I managed to speak to somebody who had had postpartum psychosis, and she’d had it twice… suddenly I thought, you know you can, somebody, somebody else has got through this”* (Woman 13). *“It wasn’t just me being a bad mum or a failure. Other people have had the psychotic, same type of thoughts and feelings that I’ve had. It wasn’t just me being mad or.. it made me realise that I was very poorly and it wasn’t my fault and I have got better and I got through it”* (Woman 2).	
**2.3: Validation – Recognising and responding to the emotional impact**	
*“You know they would never say to a woman that had been attacked ‘well that’s no big deal’ but people will separate psychosis, because to them it’s not real, to them it never happened, but it did happen to me, something was coming up my hill, it was going to kill my kid and if I didn’t kill myself he was going to die. You know it’s as simple as that, I really believed my baby was going to die and be killed”* (Woman 7). *“I said to her [GP], you know, I can’t remember the first three years of my daughter’s life, and she said, well, that’s a grief that you will just carry with you, and I thought, actually, that’s really nice, that somebody acknowledged that”* (Woman 10). *“I was worried about [partner] and how he was coping because it was, it was a LOT to deal with and umm… so they [MBU staff] spoke to him about how he was and they’re really caring of the families of people who are suffering on the ward and that, that meant a lot.. umm and it means, it stops me worrying, because I could, they could say to me, whether it was true or not, they could say ‘we’ve got them, don’t worry, you don’t need to worry about their emotions and their needs, worry about you’ and I think that was really helpful”* (Woman 8).	
**Subtheme 2.4: Making sense and processing**	
*“But it [EMDR therapy] was the key to be able to help me put that back into my longer-term memory and, and to get over all of those things that had happened”* (Woman 9). *“I really wanted to speak to someone, and I think that’s the problem, I wanted to speak to somebody that kind of under, maybe understood a bit more, or, that counselling type of thing, I don’t know whether that was the best thing to do, but, obviously a lot of times when you’re ill, it’s more about getting the medication right, and not actually… talking about what’s gone on”* (Woman 13). *“We’ve spent a long time talking about things like that. I think that’s important, because obviously she wants to. I know she won’t remember it, but she at least wants to know how, her son was born, you know and that, it’s such a huge milestone in your life, how would you not want to know”* (Family member 21).	
**Subtheme 2.5: Managing changing relationships**	
*“So my family, have found it VERY hard to understand my illness, and, er, and, (sigh) and the impact, so for the, the sort of, er, ripple effect, if you like, that goes on after your illness and during your illness is really strong”* (Woman 10). *“Since she’s been out [of the MBU], we’ve been very careful about discussing it because we don’t want to upset her, by umm, bringing back the memories of being in there”* (Family member 14). *“I think, ye maybe like a family education, so to be able to go look, we now recognise she’s at this particular stage, that means that she’s LOADS better, you know, she’s only just going through this last phase to make sure she’s completely got it out of her system, but you can now start to allow her that freedom to spend a bit of time on her own and all that kind of stuff and they can then start to step away a bit, like feel a bit of relief for them”* (Woman 6).	
**Theme 3: Planning for the future**	
**Subtheme 3.1: “Too good to be true”**	
*“I think the issue that I’m going to hit with it, is the, I’m only under the care of the perinatal team until my daughter’s one and because I was, because my baby was older when I had my psychosis and I’ve had a very long recovery time, that’s going to happen quite soon, and so that is one the limitations of the, of the service here, something, I know I’ve heard them.. talk about. They’re considering whether stopping at one is the right time, but for me that is going to be a real barrier going forward for my recovery”* (Woman 4). *“I mean I know the older you get you don’t need as much [sleep], but to her, it’s like, a major thing, ‘cos it makes her, she’s frightened of it making her psychotic….. But, I’ve tried to get over to [Daughter] it’s no big deal, but I have to be careful, because she’ll sort of say to me, “well it’s alright for you, you don’t have to do anything if you don’t want the following day”, which is true… erm, and I’ll say, yeah, but I’m trying to say to you, “don’t let it, you know, take over your life, don’t let it in, don’t let it be, get the better of you”* (Family member 16).	
**Subtheme 3.2:** ***“It lives with you”***	
*“The perinatal psychiatric team saw her throughout her pregnancy and she was given, erm, medication on the night of delivery, and luckily it didn’t happen again, I mean she still at times has, er, periods of anxiety, but whether that would, have happened anyway even if she hadn’t have had postpartum psychosis is something we, we don’t know, but we won’t ever know”* (Family member 19). *“I’ll obviously start talking to [child] about it, and… some point down the line, umm then yeah, may be, there may be moments again where I’ll need to… find out a bit more information about what happened to me, umm because, it would be important for xx to know”* (Woman 5). *“I think if I’d heard of it, I would probably have thought twice about having children myself (laughs) it’s so horrific, you know. Err I was, it was quite a surprise when she said she wanted another baby, I must admit.. ‘cos I thought, God, surely you don’t wanna go through any of that again, she said, “oh well it’ll all be monitored” and everything else, well it wasn’t done very well. I mean she was put on something but, she could have been sorted a lot better from the beginning, there wasn’t a note at the hospital where she had the baby… It wasn’t good enough, you know, they, they weren’t informed enough I don’t think”* (Family member 18).	
**Subtheme 3.3: Contributing and giving back**	
*“I think she sort of gets, erm, satisfaction from the fact that if she could do anything to stop somebody else having, sort of a severe problem in, in the future, then any research that can be done, anything that can be done to help, I mean she actually, erm, saw your request for help and sort of spoke to me about it and said, erm, would you be willing to do this, and she said, you know, she said, I’ve done quite a few, but would you be willing to contact, erm, this lady and sort of give her your point of view, and you know, and I said, well yes… because, if it could help some other family in the future”* (Family member 19). *“I’ve tried to answer people’s questions honestly and try and help, because if, if, say it’s one person from going through what I went through, by being a little bit more honest than maybe I would ordinarily be comfortable with then I’ll do that”* (Woman 8). *“I think it’s helped me to process... umm and I think it’s helped me not to feel like I’m alone because… I didn’t know anyone that had had postpartum psychosis so by talking about it, I found out about quite a few people… and knowing I wasn’t on my own umm and that I wasn’t going mad and it was a real thing and… umm, that we would get better, definitely, definitely helped”* (Woman 8).	
**Subtheme 3.4: Recognising internal resource and seeking acceptance**	
*“Ye, ye, ye, and more recently I can think about it much more positively, you know, because I used to think things like, “why on earth did that happen to me?” sort of thing and now, I think of some of the.. you know, it’s made me very strong I think and it’s also helped me to, maybe help other people going through it so I think of it, you know much more positively now”* (Woman 12). *“I guess in some ways, we’ve… come out a bit stronger as well, you know there are some positives you can take from it as well. We’ve lived through something and dealt with it as a couple, that a lot of people maybe haven’t or couldn’t so, you know it has, a small, you know positive in it”* (Family member 21). *“I, I just think in the grand scheme of things that we, we were, as terrible as it was, and it could have been a lot better, we are lucky that, you know xx made a full recovery, and that, you know, nothing, nothing detrimental happened to her or any of the children”* (Family member 15).	

### Theme 1: seeking safety and containment

The first theme, comprising three subthemes, captured the unexpected and extreme distress reported by women and their family members during the initial acute phase of postpartum psychosis, which typically lasted a few weeks in duration. Participants did not want formal psychological intervention at this time and reported that practical support, emotional containment and safety were and should be prioritised.

#### Subtheme 1.1: “What is wrong?”

Participants reported feeling overwhelmed, scared and confused and believed that their lack of knowledge prenatally about postpartum psychosis, contributed to their sense of shock. For instance, a partner reported that antenatal classes often painted a *“rosy picture”* (Family member 21) and did not adequately prepare women for difficulties that could arise.

Family members often sought help once they reached a crisis point and felt they could no longer manage the women’s symptoms of postpartum psychosis and associated distress. Consequently, family members reported feeling helpless and became more dependent on mental health professionals to provide education, support and guidance. In particular, participants wanted an explanation for the experience which was often achieved through receiving a diagnosis of postpartum psychosis:

*“My husband…his mother, my mother-in-law, were doing so much. They were hounding the staff in the psychiatric ward saying she needs a diagnosis like what is going on? and if she is going to a mother and baby unit, WHY? What is wrong?”* (Woman 1).

Having an explanation and label could relieve some of the worry and distress experienced. However, delays in being given an explanation and diagnosis exacerbated their sense of fear and uncertainty for the future and risked damaging relationships with health professionals at this critical time:

*“I was like you don’t even know me and yet you’re telling me that I’m going to be going home soon, well how do you know that, where’s that come from? If you don’t tell me what’s wrong, then how can you be telling me that I’ll be better in a bit”* (Woman 6).

#### Subtheme 1.2: “My greatest hour of need”

During the initial acute phase women reported extreme distress, they worried for their safety and worried about the wellbeing of those close to them, including their baby. As a result, most women were admitted to hospital at this time, where a range of experiences were reported. Women needed support from professionals they could trust in order to feel safe and cared for, which in turn provided reassurance for family members: “*But she felt safe [at the MBU] and because she felt safe, we felt safe”* (Family member 14)*.*

A few women, however, said they did not feel contained or supported during their initial contact with services, which exacerbated their distress and had a long lasting and detrimental impact on their recovery:

*“Well I think particularly the [first] 36 hours and how I was treated and the fact there was nobody in my greatest hour of need to help me... it was all very, very disempowering, I mean none of it could be helped, you know, the actual, umm condition… but umm that I felt very upset and let down by the response to what happened … I just felt that, well nobody could help me really, there was nobody there to hold me and help me when I needed it”* (Woman 9).

Similarly some family members reported a delay at this critical time to accessing the help and support required. Barriers to care included staff shortages, poor continuity of care, problems sharing information due to concerns around confidentiality, perceived lack of compassionate care and inappropriate hospital provision:

*“I felt like she was just sort of, labelled a crazy lady in A&E, that’s, that’s how I felt, and I felt like I was screaming and nobody was listening to me”* (Family member 15).

#### Subtheme 1.3: “Ploughing through fog” – Need for hope and reassurance

In the initial acute phase, both women and family described the woman’s presentation as much different to her usual self and expressed a sadness that they had missed out on the expected joys of parenthood. This extreme deviation from their usual character made it difficult for women to imagine a return to their previous wellness and resulted in feelings of hopelessness about the future:

*“She was a bit like the frog climbing out the well, she’d, you know, would climb up a bit, then slip back into the well, a bit like that, but she didn’t see that, she didn’t perceive that she was getting better for a long-time”* (Family member 14).

During this critical time, family questioned if they were doing the *“right thing”* and likened their experience to *“ploughing through fog”* (Family member 19). Due to this perceived sense of helplessness, family members’ reliance on professionals often became more pronounced:

*“There wasn’t anything that WE could do to make her better. It would be time and it would be medication and it would be the experience of the staff, that would make her better. WE could not do anything, other than to provide her with hopefully healthy snacks and umm... try and take her out and talk about something that wasn’t her illness”* (Family member 14).

Throughout this period, it was important for participants to receive hopeful messages about the future and for family members to be guided on how to respond, as well as receiving recognition of existing strength and resources. This feedback helped to alleviate some of their concerns and enabled family members to recognise the value of their role.

### Theme 2: Recognising and responding to the psychological impact

This theme comprised five subthemes and captured some of the psychological needs and mechanisms involved in recovery. It was important to participants that these needs were appropriately identified and responded to. Recovery was reported to be a slow process, influenced by the wider context including interactions with family, peers and professionals. This process however, was not linear, and sometimes featured relapse or the onset of depression.

#### Subtheme 2.1: “It wasn’t me” – Externalising the experience

In time, most participants started to view postpartum psychosis as a disorder related to childbirth which was out of their control. This illness representation helped to reduce self-blame and associated feelings of guilt that was associated with an earlier period of causal search:

*“Cos you think, well I must have done something to make, you know, make myself ill, ‘cos why would I get ill and not somebody else, sort of thing, obviously you start thinking it’s your own fault for being ill, or that you’ve done something, not done something, erm, that kind of thing, and I think it takes a while to realise well actually it’s just, that’s, it’s just something that happened, that it’s just unfortunate that it happened, but it’s very difficult to get your head round that”* (Woman 13).

Both women and family members sought to enhance their understanding by seeking information about postpartum psychosis. However, some participants, spoke of “*horrible stories”* (Woman 11) located online and this experience highlighted the importance of being signposted to credible and trusted sources. Information that was particularly valued was that which contained more hopeful and balanced messages about treatment, prognosis and recovery.

#### Subtheme 2.2: “Somebody else has got through this”

Participants highlighted the value of connecting and relating to others with similar experiences, which helped to dissipate feelings of isolation, normalise their experiences and provided “*tangible [evidence]”* (Family member 21) that recovery was possible:

*“I think it’s helped me not to feel like I’m alone because... reading things from umm, action on postpartum psychosis [APP] and talking to the other women on the ward where I was, we all had different things, some people had had psychosis, and knowing I wasn’t on my own umm and that I wasn’t going mad and it was a real thing and... umm, that we would get better, definitely, definitely helped”* (Woman 8).

Most women described that they needed to relate to other women with mental health difficulties prior to connecting with mothers or peers more generally. For some, this need was driven by their underlying perception or belief of them being a *“bad mum or a failure”* (Woman 2) and an associated fear of judgement. Many participants had not experienced mental health problems prior to postpartum psychosis and self-stigma was reported to be a barrier to accessing peer support. As one woman (Woman 11) highlighted: *“I had such a stigma ABOUT MYSELF, even ABOUT MYSELF having it [postpartum psychosis]”*. By comparing themselves to others with similar lived experience, women were able to recognise and address their own stigma, and in time they were able to disclose some of their experiences which facilitated the process of adjustment and acceptance to their altered view of self.

#### Subtheme 2.3: validation – Recognising and responding to the emotional impact

Following the acute psychotic phase, women said they were able to rationalise that many of the events they recalled were not accurately based in reality. Nevertheless, the emotional response associated with such traumatic events often remained with them far beyond symptom remission:

*“At the time, it felt that those things were really happening, that I felt that I really did experience the death of all of my family members and err... I wouldn’t have been able to speak like this about that, you know maybe even a year ago, I don’t know. Umm, err, it was hugely traumatic”* (Woman 9).

Many women sought psychological input to cope with the emotional impact of postpartum psychosis. Participants valued health care professionals who recognised and validated their experience. However, some women reported significant delays in receiving help and spoke of their frustration towards services which they felt did not incorporate the postpartum aspect or were not equipped to respond to a trauma, associated with a psychotic episode:

*“CMHT’s you know, you don’t fit this tick box now, you don’t fit that tick box. But you are left with this life that is destroyed.... I needed, I needed something, I needed some kind of psychological support. I needed trauma therapy, I needed somebody to understand that these things were REAL TO ME”* (Woman 7).

Participants wanted a more individualised approach that incorporated their specific emotional needs, whilst also considering their needs as a parent. To facilitate psychological input, women valued practical considerations, including provision of childcare, flexibility around timing and an option for home visits. Furthermore, women found it helpful to know their family were also being supported, because this enabled them to focus on their own emotional needs and prioritise their recovery.

#### Subtheme 2.4: making sense and processing memories

Participants’ memories during the acute period of postpartum psychosis, including their hospital admission, were often described as being extremely distressing and associated with a perceived loss of control over their lives:

*“I remember this, she wasn’t a very nice nurse that was on there and she seemed to take great pleasure in telling me that I was sectioned and I, and I, just, it really sort of frightened me so much when I heard that, umm and I think that was the horrible thing, it was like the power and control was taken away from [me] completely”* (Woman 12).

Many women felt they had behaved in ways which represented a significant departure to their usual self, which was difficult to process and make sense of:

*“I just unravelled as a person and was needing 24-hour support, for months, probably about 6 months really. Umm err and that I couldn’t enjoy my children because of it, you know, like I lost all of that really, I lost the ability to breast feed, all manner of things, more than just, and the shocking, the shocking departure out of my own home with police and ambulance and the whole street out, you know like, it’s very traumatic to process, particularly you know, if you’re able to, you know, have been, quite, you know, well-functioning up until now”* (Woman 9).

Participants spoke of events in their lives that acted as reminders of these difficult memories, including their child’s birthday, attending hospital appointments and messages on their social media accounts. These memories evoked feelings of fear, sadness and guilt and remained with women for subsequent years, highlighting the profound and long-lasting impact of postpartum psychosis. Further contributing to their distress, women reported significant gaps and uncertainty around which memories were ‘real’ and which ones were part of their psychosis. Most participants said they needed time to talk through, make sense and process these memories on both a formal and informal basis. However, some women reported having had few opportunities for this with healthcare professionals and believed the emphasis was often on *“looking forward”* (Woman 5) or *“getting the medication right”* (Woman 13).

As women actively tried to cope with their emotional responses, their strategy use evolved over time, from rumination and avoidance of reminders (e.g., by moving house, deactivating social media), to actively seeking to change their relationship with the past; for example, by focusing on their achievements. Women also sought to fill in their gaps in memory; for example, by reviewing their medical notes, writing down their experiences and spending time with family to create a timeline of their experiences. By utilising these strategies to process their experiences, women were able to develop a narrative of the events, which helped to enhance their understanding and ability to cope.

#### Subtheme 2.5: managing changing relationships

Throughout their accounts women and family members highlighted the importance of their relationships within the family to help facilitate recovery. These relationships were reported to evolve over time, in response to the multiple and often changing needs of women recovering from postpartum psychosis. In the early stages some women described feeling overwhelmed and questioned their ability to cope on their own or meet their baby’s needs. As a result, family members often took time off work to provide the practical and emotional support required:

*“They [family] were totally invaluable, we wouldn’t have been able to still, be functioning as a family if they hadn’t have dropped everything at that time”* (Woman 9).

In time, women sought to regain their independence and develop a routine which meant they needed less intensive support from their family. This was often difficult for family members who were worried about the risk of future relapse and sometimes became overly protective as a result. Participants valued professional input, including psychoeducation to help bolster the family members’ confidence and alleviate some of their worries. In particular, women valued input with regards to expected milestones and guidance as to when the family could *“step away”* (Woman 6); thereby promoting the women’s recovery and development of self-efficacy.

Further along in the women’s recovery, different coping styles were reported. Family members often believed they had to *“move on”* (Family member 14) and therefore avoided talking about the experience of postpartum psychosis. This approach sometimes raised further challenges for women who wanted to talk about their experience as a means to process and understand it. These different coping styles highlight the complexity and evolving needs within the familial relationships. Both women and family members reported joint therapy would be useful at this stage in order to openly talk through these concerns together and consider the best way to realign themselves within their relationship.

### Theme 3: planning for the future

The final theme, comprising four subthemes, related to the long-term impact of postpartum psychosis and consideration as to how to maintain and build on the progress achieved during the earlier stages of recovery.

#### Subtheme 3.1: “Too good to be true”

Due to fear of a subsequent episode or relapse, women and family members reported ongoing monitoring for symptoms or fluctuations which resulted in possible hypervigilance. As postpartum psychosis had occurred *“suddenly”* (Woman 4) and with *“no indication”* (Woman 5), women reported that their confidence had been affected and they questioned their ability to detect fluctuations in their wellbeing:

*“One of the things that was really bothering me, was when [partner] would say things like ‘are you feeling ok?’ if I was saying something, or you know, just look at me, really concerned, sort of, yeah just, it just made me feel, like it would always, like really shook me, because I’d be like ‘oh gosh, am I not ok?’, because I had no, no, umm self-awareness before anyway, it made me just think ‘OH MY GOD, maybe something’s wrong with me again’ and I just can’t even tell”* (Woman 11).

Participants often worried about the future and believed they needed additional long-term support from mental health services. In particular, those who had received input from a specialist community mental health team reflected on how difficult they had found their discharge from services at one year postpartum:

*“So the first year you’re given all the support... and then after that, where I believe that every single person that I have ever spoken to with postpartum psychosis, says yes, that first year is hideous, but WHAT about that second year”* (Woman 7).

These concerns were associated with the feeling that it was *“too good to be true”* (Family member 15) and increased awareness of the possibility of subsequent relapse. Participants emphasised the need for ongoing professional input, akin to relapse prevention and believed that they needed to be more informed in order to prepare for the future management of their mental health.

#### Subtheme 3.2: “It lives with you”

Most participants reflected on how the experience of postpartum psychosis had influenced their decision-making regarding subsequent children. Some chose not to become pregnant again, due to the increased risk of a subsequent episode and concerns for the women’s wellbeing. Whilst others felt they needed a more positive birth experience and believed they were more equipped to cope: *“it shouldn’t ever get as bad [again]”* (Family member 16). Decision making was reported to a complex process that evoked a range of difficult emotions:

*“It lives with you… for a long time, and then obviously we’re thinking you know, potentially about more kids, and then well, is it going to come back and you know, so, it affects more than just you’re ill and you get better from it… you’ve got to deal with this for the rest of your life”* (Family member 21).

For some, subsequent pregnancies facilitated a more open dialogue between women, family members and professionals about the potential risks and how to minimise these, which was described as *“healing”* (Woman 2). However, others believed they had been let down by professionals and given the *“wrong advice”* (Woman 5). Being provided with accurate and timely pre-pregnancy consultation was considered important because it provided an opportunity to discuss any concerns and helped to alleviate some of the stress and worry experienced.

#### Subtheme 3.3: contributing and giving back

A common theme in women’s recovery related to sharing their story and trying to turn their experience *“into something good for someone else”* (Woman 10). Women achieved this in different ways, including sharing their story online, attending talks, visiting MBUs and offering peer support. This often required women to be more open and honest than they would naturally be, but they reported to be driven to do so in order to help others. As one family member described:

*“If something positive can come out of it, it’s, it’s done good, hasn’t it, if, you know, she’s not gone through it in vain”* (Family member 16).

Helping others and raising awareness also enhanced women’s recovery because it enabled them to talk through and process their experience, promoted connection with other women with similar lived experience and helped individuals to feel as though they had re-gained control over their lives:

*“How do I make sense of it now, I do all the, I raise awareness, I do the talks because I had no control, I had no power and because of the area in which I live, and only because of where I lived, I believe I lived. Had I have been in a different postcode without a specialist team I wouldn’t be alive and [that’s] how I gained control over that” (Woman 7).*

#### Subtheme 3.4: Recognising internal resource and seeking acceptance

Over time, women demonstrated more compassion and acceptance towards themselves. To achieve this, women needed to adjust to their new identity; both as a parent and as someone with lived experience of postpartum psychosis:

*“I just used to really miss, how I used to feel… I felt like I was quite a confident person and... umm nothing phased me and... I just felt like I wasn’t that person anymore and umm… in more recent years I’m closer to my old self than I ever have been, but umm, but I suppose I have to accept that I’m never going to feel like that again”* (Woman 12).

Many participants reflected on the inner strength they had demonstrated and many highlighted positive changes as a result of their experiences. This included renewed appreciation for their family, increased *“compassion and understanding for mental illness”* (Woman 2), enhanced self-belief and gratitude for their child. Many used the word *“lucky”* when describing their experience and were motivated to live life to its fullest:

*“I had this massive, massive moment where I knew I was back and some, and I looked around and I watched all my family laughing and my daughter, she had a couple of her friends and there were her cousins, all mucking about and I thought ‘my god’ this would never have happened if I had died… But because I had good treatment, I looked around and just thought I am the luckiest person in this world”* (Woman 7).

This recognition of renewed strength and resilience was important for many participants and enabled a more optimistic outlook for the future and enhanced their self-belief regarding their ability to cope.

## Discussion

The aim of this qualitative study was to explore and understand the psychological needs, experiences and preferences for psychological intervention for women from the perspective of women and family members. Consistent with previous research (e.g., [16, 28, 35]) the themes identified in this study highlight that recovery is a complex and active process, in which women’s needs change over time. The findings illustrate a range of psychological needs that have to be met to facilitate change and promote wellbeing; which could be enhanced through psychological intervention.

As previously reported, the results reiterate the importance of family support in recovery from postpartum psychosis as well as highlighting the potential strain this experience can place on relationships [27, 45]. The process of adjusting to the changing needs within their relationships brought about challenges often associated with fear of further relapse. By working through these difficulties and attempting to rebuild their relationships, strength was often identified by participants. This is comparable to findings from the posttraumatic growth literature in which it has been found that positive psychological changes, including improved interpersonal relationships and an increased sense of personal strength, can result from the ‘struggle’ with highly challenging life events (p. 6 [46];). This finding can be compared to that of psychosis, in which there is a substantial evidence base regarding the use of family intervention to enhance coping skills and reduce stress [23, 25]. Family interventions typically focus on improving the interpersonal environment and may include problem solving and goal setting. Given the similar behavioural patterns, this warrants further investigation in the context of postpartum psychosis. Due to the stressful nature of supporting a relative with postpartum psychosis [14, 18] family members may also benefit from support for themselves and potential barriers to accessing family support should be considered, including a reported need for family to appear ‘strong’ [16].

During the latter stages of recovery the importance of psychological support and intervention was identified in this study. The emotional impact of postpartum psychosis and associated sense of loss remained with women for many years. Women’s ability to recognise strength, change their perspective and develop acceptance towards their experience was important for recovery, but this was experienced as a lengthy process albeit one often enhanced through the support of others. Many of the sample had no prior mental health difficulties and viewed postpartum psychosis as a significant shift from their usual functioning. Women needed to process and make sense of their experiences before they could successfully transition and accept their altered self-identity, as both a mother and someone with lived experience of postpartum psychosis. This replicates previous findings that women need to integrate their ‘dual identity’ following a severe maternal mental illness (p. 17 [47];). When this need was not met, some women described feeling invalidated and unable to move on, resulting in processes of rumination, subsequent depression and avoidant behaviours.

Furthermore, some individuals described being traumatised by their experience and reported re-experiencing distressing memories. Indeed, one participant required specific treatment for traumatic experiences (EMDR). The potential for individuals to perceive their experiences as traumatic should be explored on an individual basis particularly if there has been a traumatic childbirth experience, because this is associated with the development of Post-Traumatic Stress Disorder [48]. Drawing on the wider literature and recommendations it is possible that CBT for trauma may be beneficial by enabling individuals to confront their distressing memories and develop more adaptive ways of coping [49]. However, further investigation is required before any conclusions can be drawn.

The majority of women (94%) in this sample were admitted to hospital for treatment and many of these admissions were involuntary under the Mental Health Act (MHA [50];). Inpatient care is recommended by NICE [8] in order to provide immediate treatment and to ensure the safety of the mother and the baby. However, many participants reported prominent and distressing memories of their hospital admission. Negative consequences associated with involuntary hospitalisation have been recognised for other mental health disorders, including stigmatisation, impaired self-efficacy and self-esteem [51, 52] and subsequent reluctance to seek outpatient help [53]. Exactly how these findings compare to that of postpartum psychosis needs further investigation. However, given the increased stigma associated with being a mother and having a serious mental health disorder [47, 54], alongside perceived powerlessness [16], this experience is likely to be extremely stigmatising for new mothers. It is crucial that this is managed well and professionals are perceived as being caring and supportive.

Another prominent issue was regarding the future risk of another postpartum or non-postpartum episode, such as anxiety or depression, which has been found to be more prevalent following postpartum psychosis [15]. It was important for participants to be provided with information about possible symptoms and ways to manage; however, some individuals reported hypervigilance and fear. Participants clearly requested accurate and balanced messages regarding the future. It could be helpful to draw upon the psychosis literature, in which there is emphasis on ‘staying well’ through psychoeducation and the development of a relapse prevention plan, which can help to improve understanding of understanding of triggers and early warning signs [55, 56]. Developing a relapse prevention plan with women and/or family members may therefore help to enhance their understanding and promote wellbeing.

### Strengths and limitations

These findings should be considered in the context of some methodological limitations. All participants identified as White British and most were well-educated professionals with minimal prior mental health difficulties. The homogenous nature of this sample may limit the transferability of findings and the outcomes may underestimate the difficulty in accessing appropriate services. For example, it has been found that black and minority ethnic groups have disproportionality high rates of psychosis, yet experience more adverse pathways into care [57] and report delays in help-seeking and acceptance of diagnosis [58]. Women and families with different backgrounds to the current sample may therefore have different needs which may require further exploration before any conclusions can be drawn.

Furthermore, all family members reported being actively involved in the women’s recovery and had advised the woman of their involvement in this study, which may be indicative of a more open and trusting relationship. Given the prominent role family was ascribed throughout the women’s recovery, there may be implications for women without such a supportive family network, or relationships in which individuals did not feel able to openly discuss their experiences. As all participants actively contacted the researcher to participate in the research, the results may be biased towards those women and family members who were willing and actively wanted to discuss their experiences. The outcomes may therefore underestimate the level of avoidance found more generally across this population. This finding may have implications for psychological services and engagement with service users, alongside implications on recovery styles. For example, higher levels of self-disclosure following a first episode of psychosis were found to be associated with higher levels of post-traumatic growth and recovery [59].

The data corpus was rich coming from women and family members who had high levels of information power [60]. This enabled thematic saturation to be achieved [61] and data collection ceased accordingly. A maximum variation sample was achieved, in which participants were successfully recruited from across England and Wales, with previous, subsequent, or no other pregnancy or mental health diagnoses reported. The heterogeneity across the sample in relation to time since onset and experience of service provision received is viewed as a strength, because it allowed for a diverse range of perspectives during all stages of recovery to be captured. It is recognised, however, that the types of intervention received by some participants may not reflect current management and treatment approaches; for example, there is now increased access to specialist perinatal services and MBU provision in the United Kingdom [62]. Although the reported time since onset was up to 23 years, the majority of participants (*n* = 19; 90%) reported on experiences that occurred within the last 10 years and the mean length of time across the sample was found to be 5½ years. Whilst recall in relation to childbirth and traumatic life events, including psychosis, is generally reported to be excellent [35], it is possible that the length of time since onset may have impaired the accuracy of participants’ recall.

### Clinical implications

The findings of this qualitative analysis illustrate the complexity of women’s recovery and suggest that psychological approaches have been and would be beneficial for women who have experienced postpartum psychosis. The themes illustrate a temporal element, in which women’s psychological needs change over time. In the acute phase, emphasis should be placed on providing a safe and suitable environment, building trust and providing access to specialist perinatal services. Beyond the acute phase, women need to feel connected, re-establish their relationships and be provided with the opportunity to process their experiences and associated sense of loss. Psychological interventions were sought at this time and are recommended to facilitate this adjustment process. Finally, as women seek to integrate their experiences, they may benefit from support to maintain their wellbeing, manage fear of relapse and plan for their future. Psychological approaches deemed useful are those incorporating acceptance or compassion based techniques, family interventions, trauma work and relapse prevention planning. Table 5 provides an overview of when such interventions could be utilised to facilitate and enhance recovery.

**Table 5:** Clinical implications for managing postpartum psychosis and facilitating recovery

Subtheme	What is needed?		How should this be delivered?	
	Women	Family	Professionals	Service/policy
**1. Seeking safety and containment**				
**“What is wrong?”**	Prompt assessment and recognition of postpartum psychosis (PP)	Support to boost their understanding of PP, signposted to credible information, such as APP literature	Complete timely assessment and provide factual information regarding prognosis once diagnosis is made	Specialist training for staff in how to respond and manage PP and increased awareness e.g., through antenatal classes and midwife
**“My greatest hour of need”**	Emphasis on feeling safe and supported. Ideally inpatient care provided in an MBU	To be involved in decision making and informed of treatment plan	Emphasis on building a relationship and devising a care plan	Need for local MBU provision and specialist community mental health team input
**“Ploughing through fog” - Need for hope and reassurance**	To be given optimistic and realistic messages about the future	Existing strength and resource recognised and utilised within the family	Need to promote hope, drawing on prognosis literature	Clear clinical pathways. Clarity about how to involve family
**2. Recognising and responding to the psychological impact**				
**“It wasn’t me” - Externalising the experience**	Support to develop a balanced understanding of PP	To enhance women’s understanding e.g., through own reading and involvement in acute phase	Develop psychoeducation with woman and family. Help to externalise experience and reduce self-blame	Ensure provision of specialist knowledge available in services e.g., through perinatal team
**“Somebody else has got through this”**	Connecting with peer networks to help normalise experiences	Linking to other family/partners, share experiences and coping	Help to build up confidence and address potential barriers to peer connection	Develop links with wider peer networks e.g., APP
**Validation – Recognising and responding to the emotional impact**	Emotional impact of PP recognised, utilise both formal and informal support	Family consider their own emotional needs e.g., liaising with MBU staff, GP and personal networks	Complete a flexible and holistic assessment, drawing on biological, psychological and social aspects	Ensuring streamlined clinical pathways, including increased access to psychological therapies
**Making sense and processing**	Have someone who is knowledgeable about PP to talk to and to make sense with. Allow time to do this	Be guided by the woman e.g., if they want to create a timeline, fill in gaps, then support this process	Recognise long term impact. Promote techniques to enhance coping skills, self-care, self-compassion and acceptance	Ensure pathways consider all areas of need, including access to psychological therapies
**Responding to changes in the relationship**	Opportunity for joint input to talk through any concerns	Professional guidance, including best ways to support, when to withdraw	Provide guidance to family, help allay their fears and anxieties	Consider family intervention, drawing on evidence-based approaches
**3. Planning for the future**				
**“Too good to be true”**	Information regarding symptoms to monitor and how to manage	Support how to respond, e.g., when experiencing increased stress	Provide relapse prevention planning – identify triggers and early warning signs	Future plan for when perinatal team withdraws one year postpartum
**“It lives with you”**	Opportunity for pre-conception counselling	To be involved in counselling, opportunity to share own concerns	Pro-actively offer advice to inform decision making	Further develop clinical guidelines re: pre-conception counselling
**Contributing and giving back**	When appropriate, utilise opportunities to ‘give back’ and share story		Facilitate involvement and incorporate into service development, e.g., developing peer support networks	
**Recognising internal resource and seeking acceptance**	Strengthening resource, facilitated in earlier stages		Utilise therapeutic approaches that draw upon pre-existing strengths – acceptance and compassion based approaches could be considered.	

### Future research

A number of recommendations regarding women’s psychological needs and areas for intervention have been recommended. Thus, future research should focus on developing an appropriate intervention and testing the feasibility, acceptability and effectiveness of this, with emphasis on the mechanisms involved and appropriate timing to offer different aspects of this intervention. Family interventions have also been recommended in order to enhance the family members’ ability to provide support for women and manage their own coping. Further research is therefore required to investigate how and when family interventions could be implemented. Future research should also focus on the impact, role of and support needed for family members.

## Conclusions

The findings of this study highlight the need for psychological and psychosocial intervention following postpartum psychosis to facilitate and enhance women’s long-term recovery and functioning. There needs to be a more robust pathway for women who have experienced postpartum psychosis which incorporates their long-term psychological and psychosocial needs. Recommendations have been provided regarding the types of intervention that would be beneficial and further investigation is needed to develop and test the effectiveness of these interventions.

## Data Availability

The datasets generated and analysed during the current study are available from the lead author on reasonable request.

## Abbreviations

APP: Action on Postpartum Psychosis
CBT: Cognitive Behavioural Therapy
EMDR: Eye Movement Desensitisation and Reprocessing
MBU: Mother and Baby Unit
MHA: Mental Health Act
NHS: National Health Service
NICE: National Institute for Health and Care Excellence
PP: Postpartum Psychosis
